# Organization of the “East Texas Medical Association,” at Nacogdoches

**Published:** 1886-12

**Authors:** J. E. Mayfielc

**Affiliations:** Secretary E. T. M. A.


### EAST TEXAS MEDICAL ASSOCIATION.

In response to a call by several M. D.'s of Nacogdoches county, a number of regular physicians from the counties of Angelina, Cherokee, Nacogdoches, and Shelby met in the town of Nacogdoches on 17th November, 1886, and organized under this name, adopting constitution and by-laws, electing officers and transacting other kindred business. The constitution names the objects to be "to foster and promote medical learning and professional qualification and brotherhood." Membership is limited only to "graduation from a regular medical school in good standing, and conformity to the code of ethics of the American Medical Association." A by-law says, "all practitioners and students of medicine are cordially invited to attend meetings and participate in discussions, and all meetings shall be held with open doors to the public." A general inclination prevailed to extend non-voting membership to under-graduates and practitioners pursuing the regular system of medicine. No fees or dues are required. The funds for necessary expenses are raised by a tax *per capita.* The association meetings are quarterly, the time and place to be fixed by the preceding meeting. The next meeting will be in the town of Rusk on the first Tuesday in January, 1887, at 7:30 p. m., and will adjourn on the next Thursday morning. New officers are to be elected at this and every succeeding January meeting.

The following is a list of enrolled members, showing officers and essayists for coming meeting: R. D. Bone, President; T. J. Murph, 1st Vice-President; A. H. McCord, 2d Vice-President; R. B. Hooper, 3d Vice-President; S. E. Mayfield, Secretary; W. G. Jameson, Assistant Secretary and Treasurer. Essayists, J. H. Barham, on Forceps Delivery; J. O. Furlow, Puerperal Septicæmia and Antisepsis; J. E. Mayfield, Typho-malarial Fever; J. A. Abney, Malarial Hæmaturia; W. G. Jameson, Surgical Reports; all members to report special cases. J. K. Sturdevant, J. H. Evans, E. B. Clements, J. T. Hoya, W. H. Campbell, W. I. Coon, G. E. Samuels, A. S. Alford, J. W. Rogers, R. B. Warren, A. M. Denman, T. S. Spivey, W. B. Treadwell, T. Y. T. Jameson, G. M. Abney, J. C. Falvey. Total memhership, 25. Several others expected to be present and join, but failed to appear. It is expected that a large majority of the physicians of East Texas will become members, and that the E. T. M. A. will be represented in the Texas State Medical Association.

The Secretary was instructed to furnish a synopsis of the proceedings to DANIEL'S TEXAS MEDICAL JOURNAL, the Texas Courier-Record of Medicine and the local newspapers with request to publish.

A vote of thanks to the profession and citizens of Nacogdoches for hospitality and courtesies was unanimously and feelingly passed.

J. E. MAYFIELC, M. D..
Secretary E. T. M. A.

